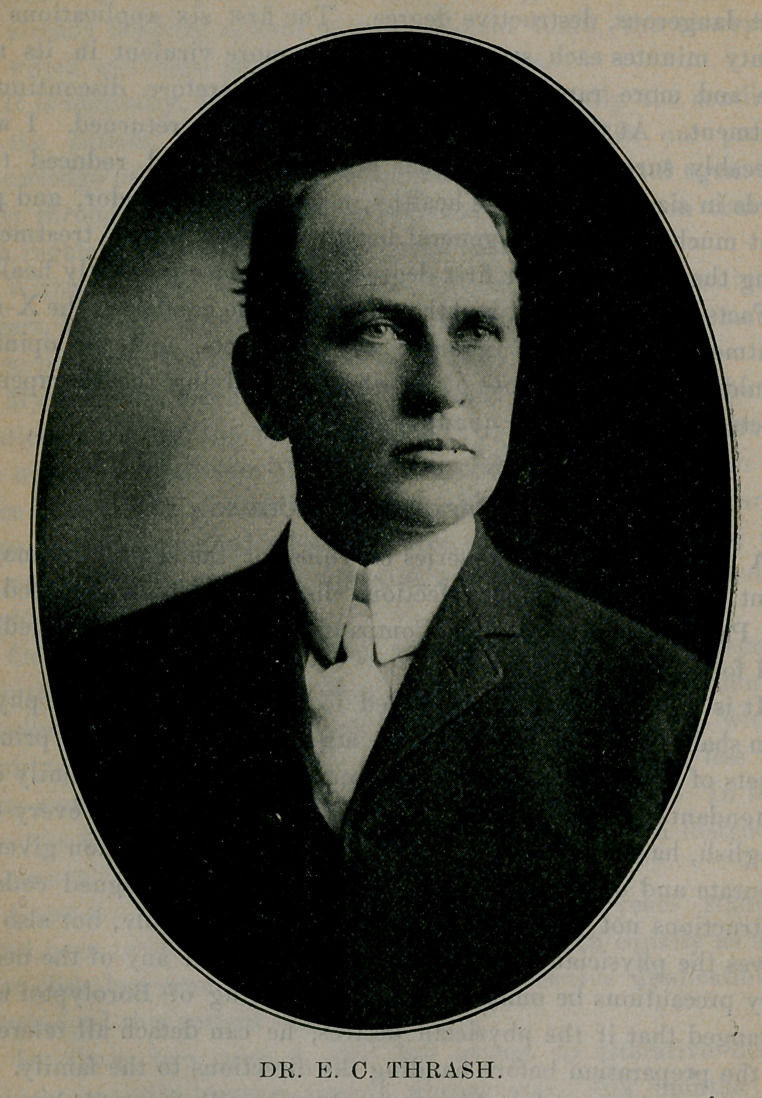# Dr. E. C. Thrash

**Published:** 1903-07

**Authors:** 


DR. E. C. THRASH.

### *EDITORIAL NOTES AND COMMENTS.*

The Business office of THE JOURNAL-RECORD is No. 64 Marietta Street.

The Editorial office is 318-319-320 The Prudential.

Address all Business communications to Dr. M. B. Hutchins, Mgr.

Make remittances payable to THE ATLANTA JOURNAL-RECORD OF MEDICINE.

On matters pertaining to the Editorial and Original Communications address Dr. Bernard Wolff, Atlanta.

Reprints of original articles will be furnished at cost price. Requests for the same should always be made *on the manuscript.*

We will present, post-paid, on request, to each contributor of an original article, twenty (20) marked copies of THE JOURNAL-RECORD containing such article.

### DR. E. C. THRASH.

DR. ELMORE C. THRASH, the second Vice-President of the Georgia Medical Association, was born on the plantation of his father, the Rev. E. C. Thrash, in Meriwether county, February 21, 1867. He worked on his father's farm until he was about seventeen years old, attending school in the vicinity during its sessions. He received his academic education at the Gordon Institute, Barnesville, Ga., and after graduation pursued the avocation of school-teacher for five years. He then took up the study of medicine under Dr. E. B. Terrell, brother of the present Governor. He completed his medical course at the University of Louisville on March 15, 1891, graduating second in a class of one hundred and fifty. His father had ten children and slender means to devote to their education, but his discipline and good training stood them in good stead in the battle of life. Imbued with the desire for knowledge, and being convinced of its utility as an element of success, young Thrash borrowed money from his friends to carry out his laudable object. After graduating in medicine he found himself burdened with debt, but with debt that was an investment which was destined to yield large returns. After some years of ups and downs, Dr. Thrash had the satisfaction of seeing himself free from financial obligation, in the enjoyment of an excellent practice and a sufficient competency.

Dr. Thrash was married to Miss Lucy Crouch in 1893.

## Figures and Tables

**Figure f1:**